# Supplementary material for: Structure–function analysis of the extracellular domain of the pneumococcal cell division site positioning protein MapZ
Source: Nat Commun. 2016 Jun 27;7:12071. doi: 10.1038/ncomms12071 (PMC4931243; doi:10.1038/ncomms12071)
Supplement: Supplementary Information — Supplementary Figures 1-12, Supplementary Tables 1-3 and Supplementary References [file ncomms12071-s1.pdf]

SUPPLEMENTARY INFORMATION

SUPPLEMENTARY FIGURES

Supplementary Figure 1

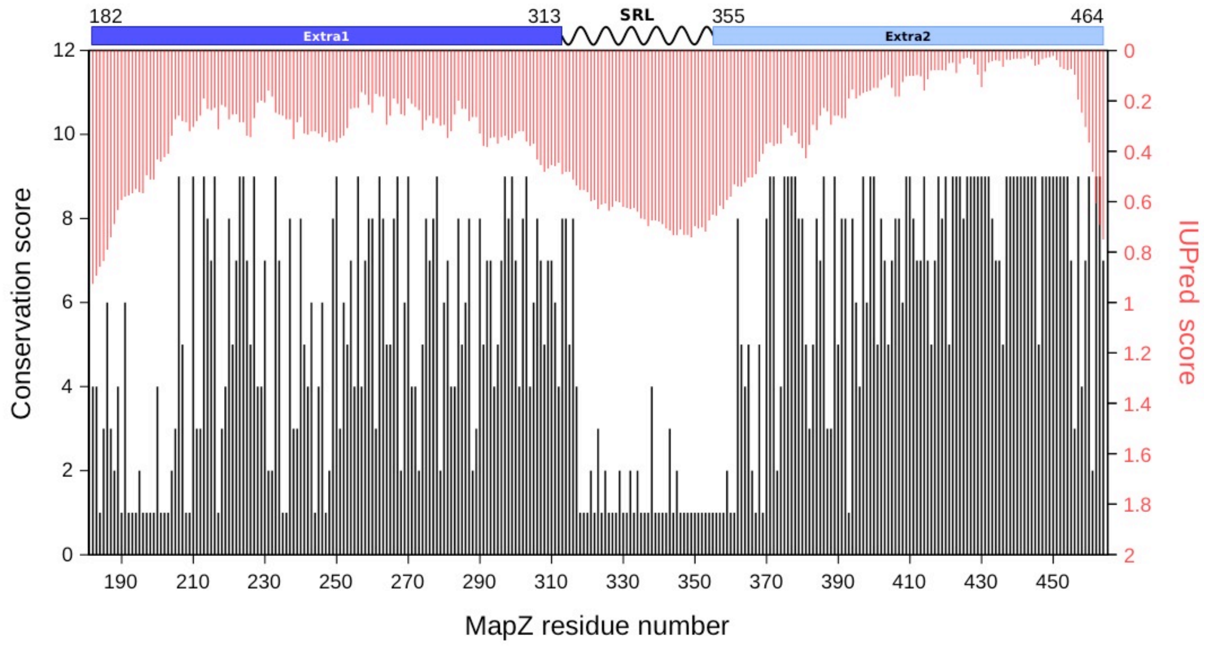

Supplementary Figure 1. Disorder of the serine-rich linker

Amino acid conservation obtained from sequence alignments on 81 protein sequences arising mainly from *streptococci* species using the Consurf webserver[1] and IUPred scores[2] are plotted in black and red, respectively. Consurf scores are ranked from 0 (not conserved) to 9 (highly conserved), while in IUPred scores are related to the predicted disordered tendency, ranging from 0 (rigid) to 1 (highly flexible). Low conservation scores indicate the less conserved amino acids in the sequence. The serine-rich linker (SRL) between the two $MapZ_{extra}$ subdomains is disordered and its amino acid sequence is poorly conserved.

Supplementary Figure 2

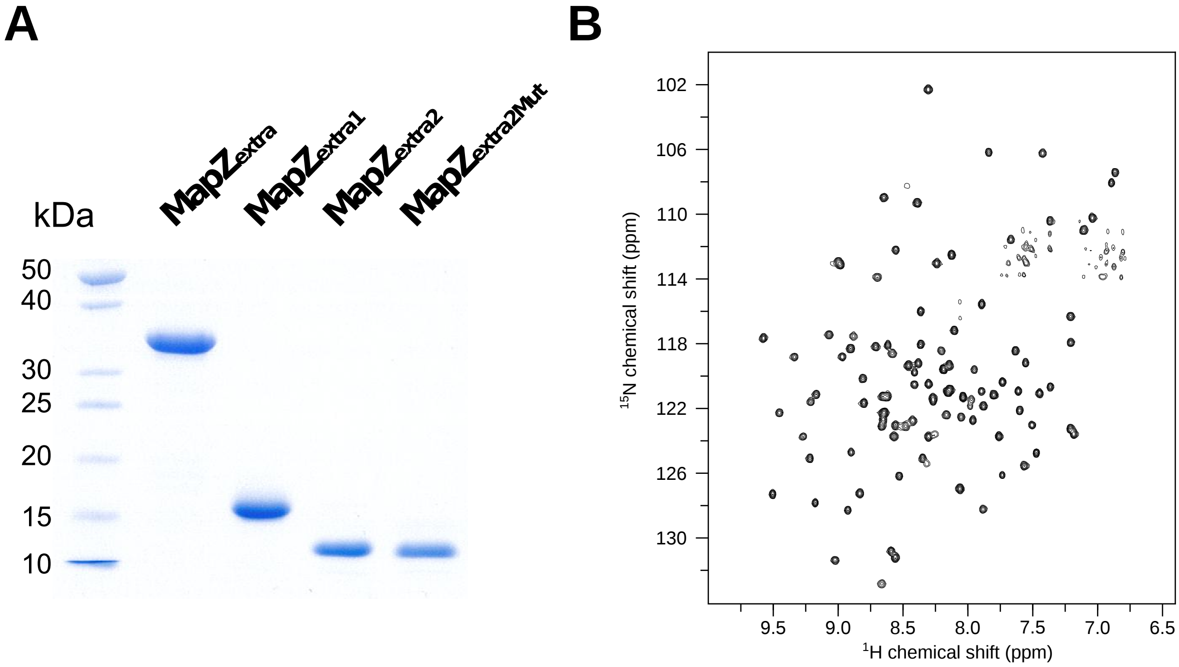

Supplementary Figure 2. Purification of MapZ$_{extra}$ subdomains and NMR spectrum of MapZ$_{extra2Mut}$

(A) The MapZ extracellular domain (MapZ$_{extra}$), the N-terminal subdomain from Q182 to G313 (MapZ$_{extra1}$), the C-terminal subdomain from S355 to Y464 (MapZ$_{extra2}$) and the subdomain MapZ$_{extra2}$ containing the following amino acid replacements R409A, Y411A, N428A, Y430F, Y450A, F451L and N454A (MapZ$_{extra2Mut}$) were overproduced in *E. coli* BL21(DE3), purified and analyzed by SDS–PAGE. (B) 2D-[^{1}H,^{15}N]-BEST-TROSY spectrum of MapZ$_{extra2Mut}$. This dispersion of resonances in this spectrum is similar to that presented in Fig. 1B for MapZ$_{extra2}$ suggesting a conserved fold of the mutated subdomain.

Supplementary Figure 3

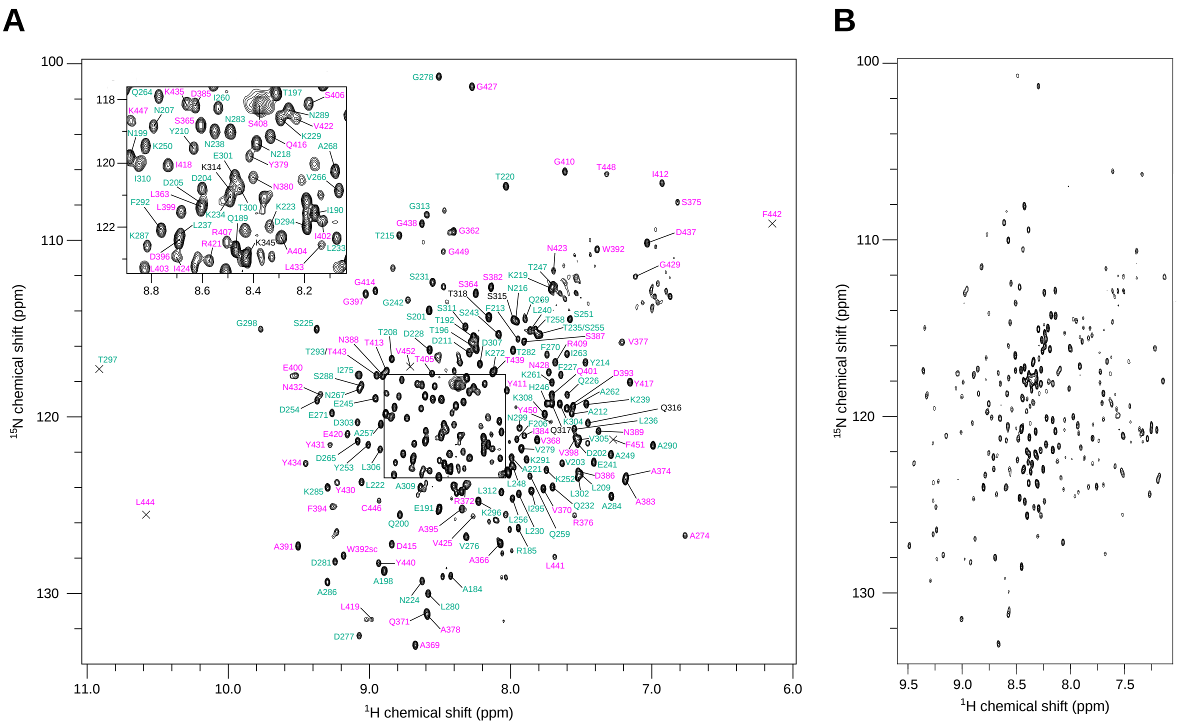

Supplementary Figure 3. Independent folding of MapZ$_{extra1}$ and MapZ$_{extra2}$ subdomains and flexibility of the SRL

(A) 2D-[^{1}H,^{15}N]-BEST-TROSY of MapZ$_{extra}$ recorded at 25 °C and pH 7.5, is displayed with the peak assignments colored in turquoise and magenta for the subdomains MapZ$_{extra1}$ and MapZ$_{extra2}$, respectively. Crosses (x) show resonances with intensities below the selected contour level threshold. (B) Region of MapZ$_{extra}$ 2D-[^{1}H,^{15}N]-BEST-TROSY spectrum recorded at 25 °C and pH 4.5. New intense peaks are detected at pH 4.5 in addition to signals from the two MapZ$_{extra1}$ and MapZ$_{extra2}$ subdomains, highlighting the presence of amide protons from the disordered serine-rich linker (SRL) in fast exchange with water.

Supplementary Figure 4

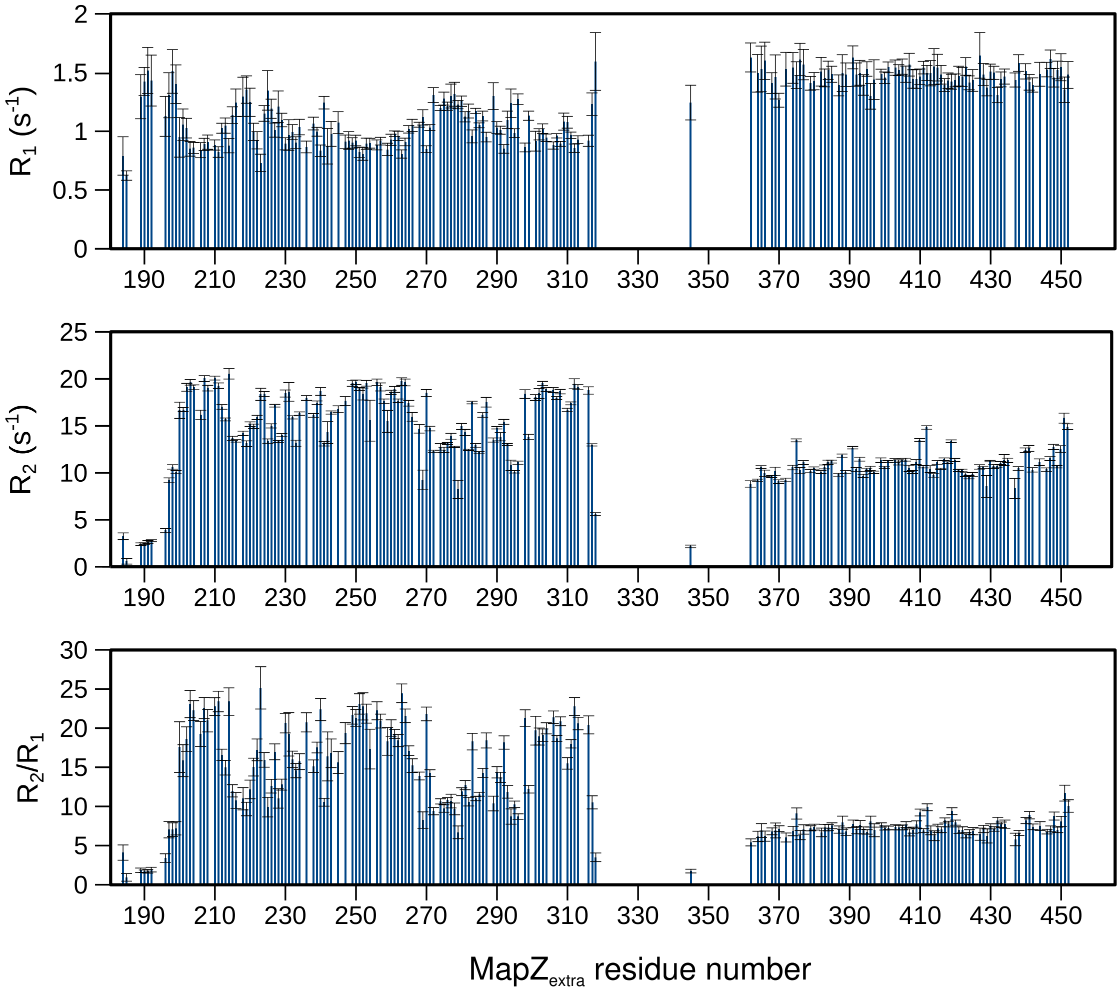

Supplementary Figure 4. NMR relaxation measurements on full-length MapZ$_{extra}$

R_1, R_2 and R_2/R_1 values, measured at 25°C, pH 7.5 and 600 MHz in full-length MapZ$_{extra}$, are represented as histograms along the protein sequence in the upper, middle and lower graphs, respectively. Standard deviations on individual values are shown as vertical segments in black. While MapZ$_{extra2}$ exhibits relatively stable and low R_2/R_1 values along its sequence, MapZ$_{extra1}$ presents higher and more heterogeneous values resulting from its anisotropic shape and flexible regions. One residue from the serine-rich linker was assigned unambiguously. Its low R_2 and R_2/R_1 values evidence a fast local motion of the serine-rich linker. Recording and processing of these relaxation data is reported in the Supplementary Methods section.

Supplementary Figure 5

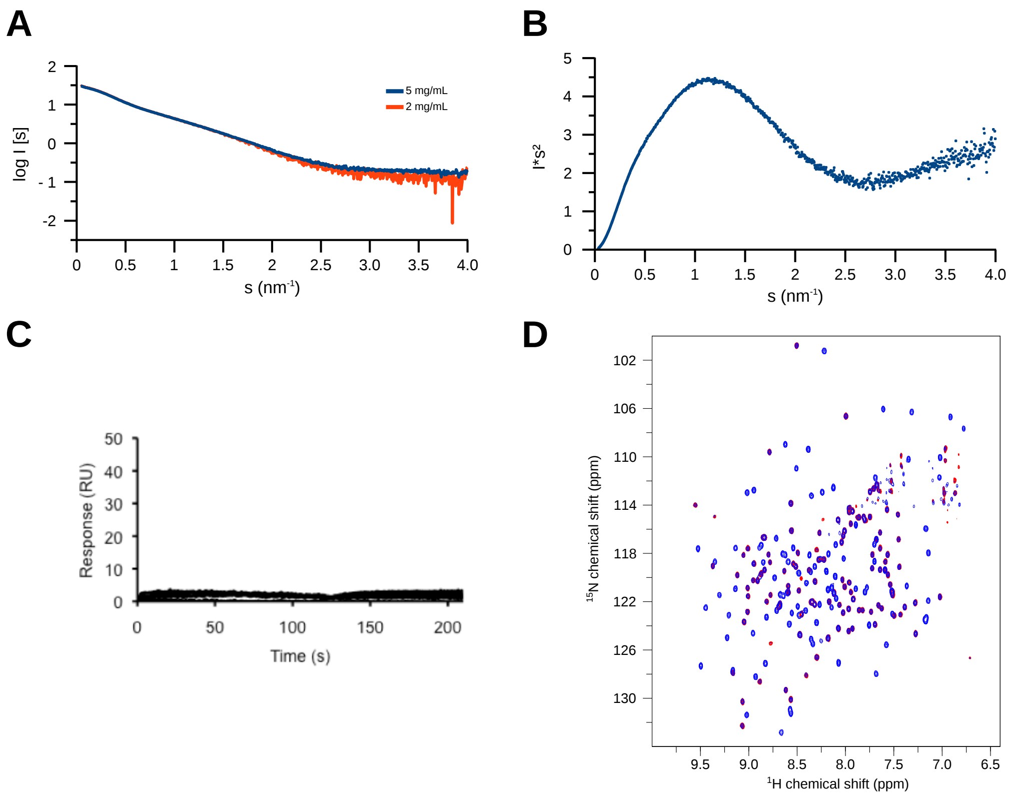

Supplementary Figure 5. Biophysical data showing that MapZ$_{extra1}$ and MapZ$_{extra2}$ are independent domains

(A) SAXS data of MapZ$_{extra}$ at 5 mg mL^{-1} (blue) and 2 mg mL^{-1} (red). (B) Kratky plot derived from the SAXS data recorded at 5 mg mL^{-1}. Its pattern is characteristic of a protein exhibiting folded domains and disordered regions. (C) SPR analyses of the interaction between MapZ$_{extra1}$ and MapZ$_{extra2}$. MapZ$_{extra2}$ was covalently coupled to the surface of a CM5 sensorchip. Increasing amounts of MapZ$_{extra1}$ were injected onto the MapZ$_{extra2}$ coupled sensorship. RU, response units. The graph is representative of experiments made in triplicate and suggests the absence of detectable interaction between MapZ$_{extra1}$ and MapZ$_{extra2}$ subdomains. (D) Superposition of 2D-[^{1}H,^{15}N]-BEST-TROSY recorded on ^{15}N-labeled MapZ$_{extra1}$ before (red) and after (blue) addition of two equivalent of ^{15}N-labeled MapZ$_{extra2}$. Absence of chemical shift variations between the two spectra confirms the absence of interaction between MapZ$_{extra1}$ and MapZ$_{extra2}$ subdomains.

Supplementary Figure 6

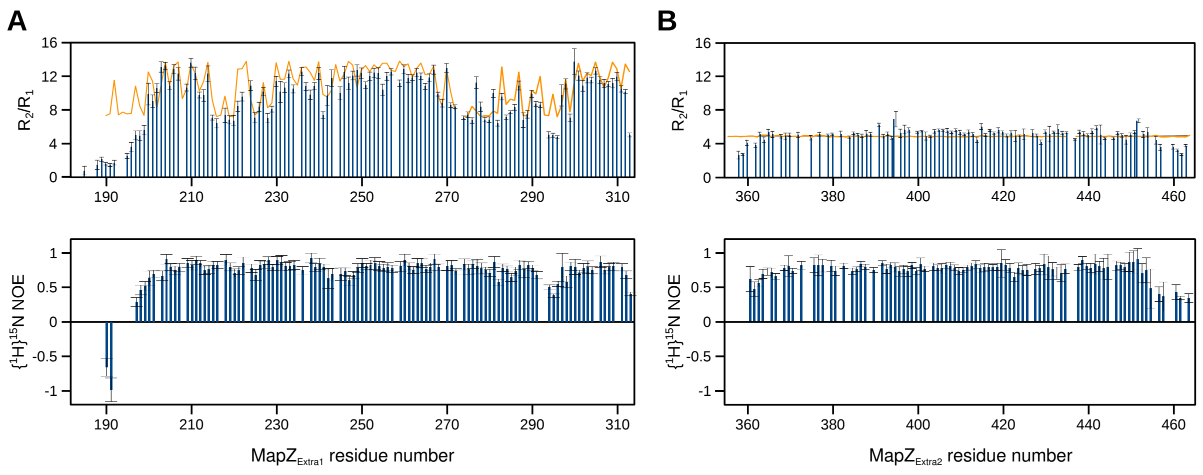

Supplementary Figure 6. Dynamics behavior of MapZ$_{extra1}$ and MapZ$_{extra2}$ individual subdomains

R_2/R_1 and *$\{^1H\}^{15}N$-NOE* relaxation parameters measured for the residues of the (A) MapZ$_{extra1}$ and (B) MapZ$_{extra2}$ individual subdomains. These parameters are shown as blue histograms as a function of the protein sequence. Standard deviation to each value is shown as a black vertical segment. R_2/R_1 values back calculated with the HYDRONMR software[3] from the lowest energy structure of MapZ$_{extra1}$ and MapZ$_{extra2}$ are shown as an orange line. Global profile of the back calculated R_2/R_1 values are representative of the difference in anisotropy between the two domains. In MapZ$_{extra1}$, the N-terminal region (residues 182 to 199) exhibits a significant flexibility. In MapZ$_{extra2}$, the 10 last residues (454 – 464) show also an increased flexibility due to their terminal position, on the contrary to the rest of the subdomain.

Supplementary Figure 7

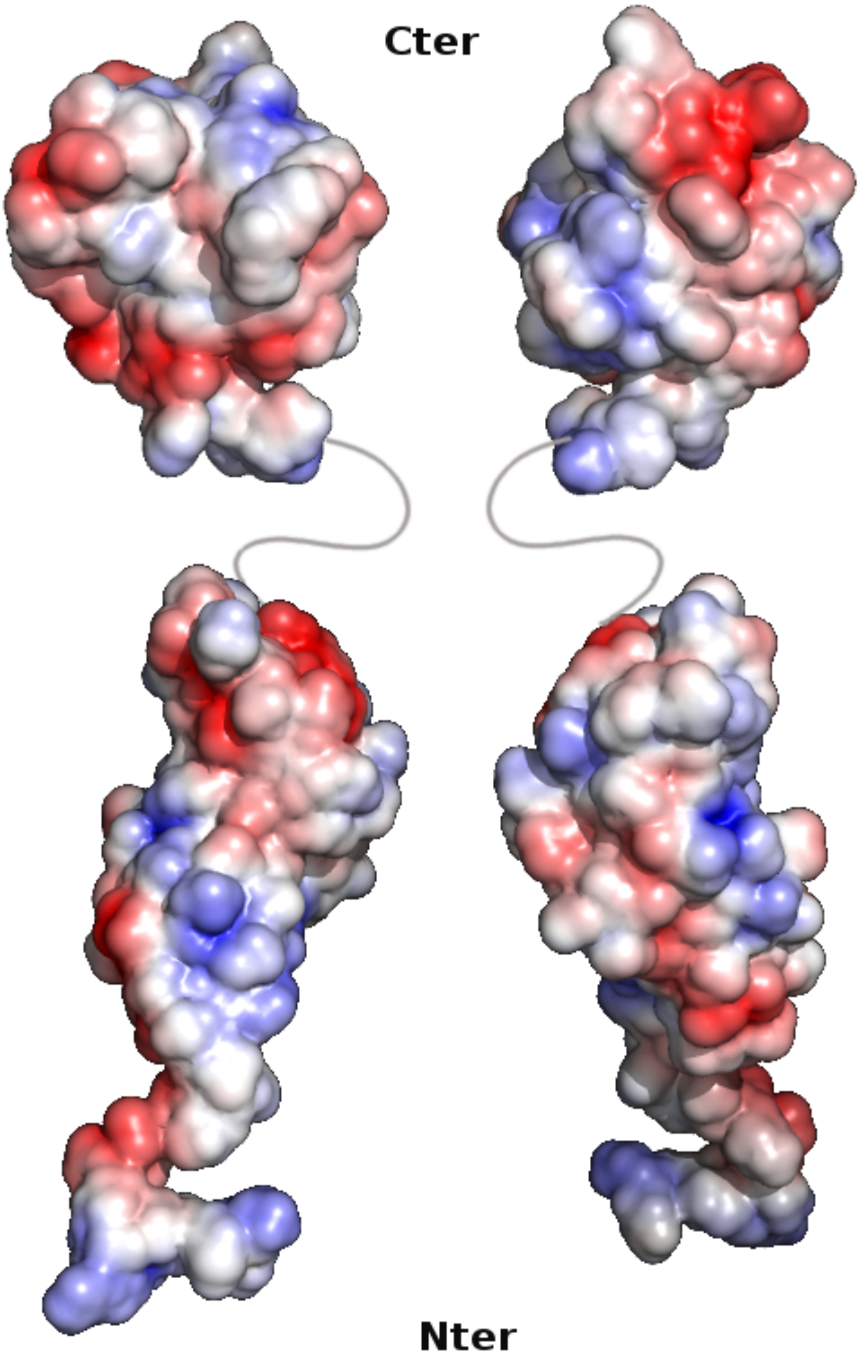

Supplementary Figure 7. Electrostatic surface representation of MapZ$_{extra}$

The MapZ$_{extra}$ structure is colored accordingly to the electrostatic suface potential, with positive and negative regions in blue and red, respectively. Left and right structures are rotated by 180° along the y-axis. Structures are represented in the same orientation as in Fig. 2A.

Supplementary Figure 8

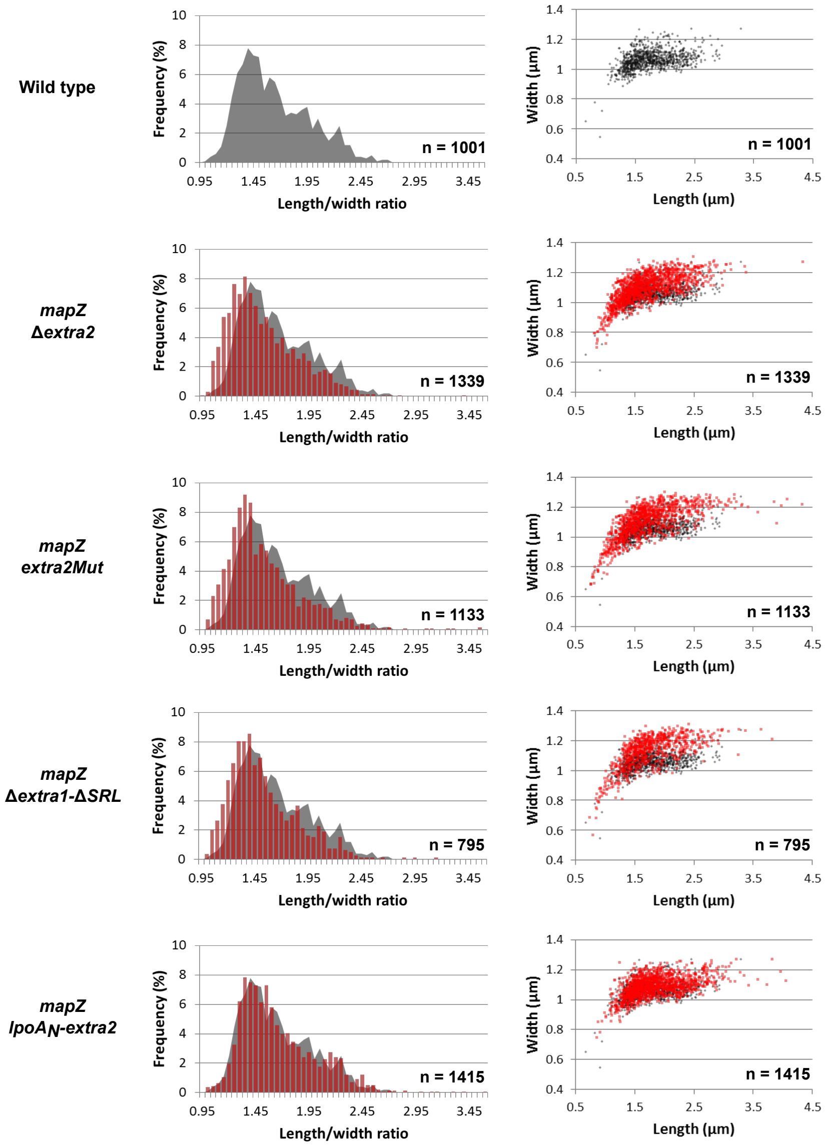

Supplementary Figure 8. Cell size parameters of exponentially growing wild type and *mapZ* mutated strains

Cell size parameters are presented either (A) as histograms showing the frequency of cell length / cell width ratio or (B) dot cloud in which cell width distribution is presented as a function of the cell length. WT cells are shown as gray shadows or dot clouds whereas *mapZΔextra2*, *mapZ-extra2Mut, mapZΔextra1-ΔSRL*, and *mapZ-lpoA$_N$-extra2* strains are shown as red histograms or dot clouds. n indicates the number of cells analyzed. Statistical analyses are representative of experiments made in triplicate.

Supplementary Figure 9

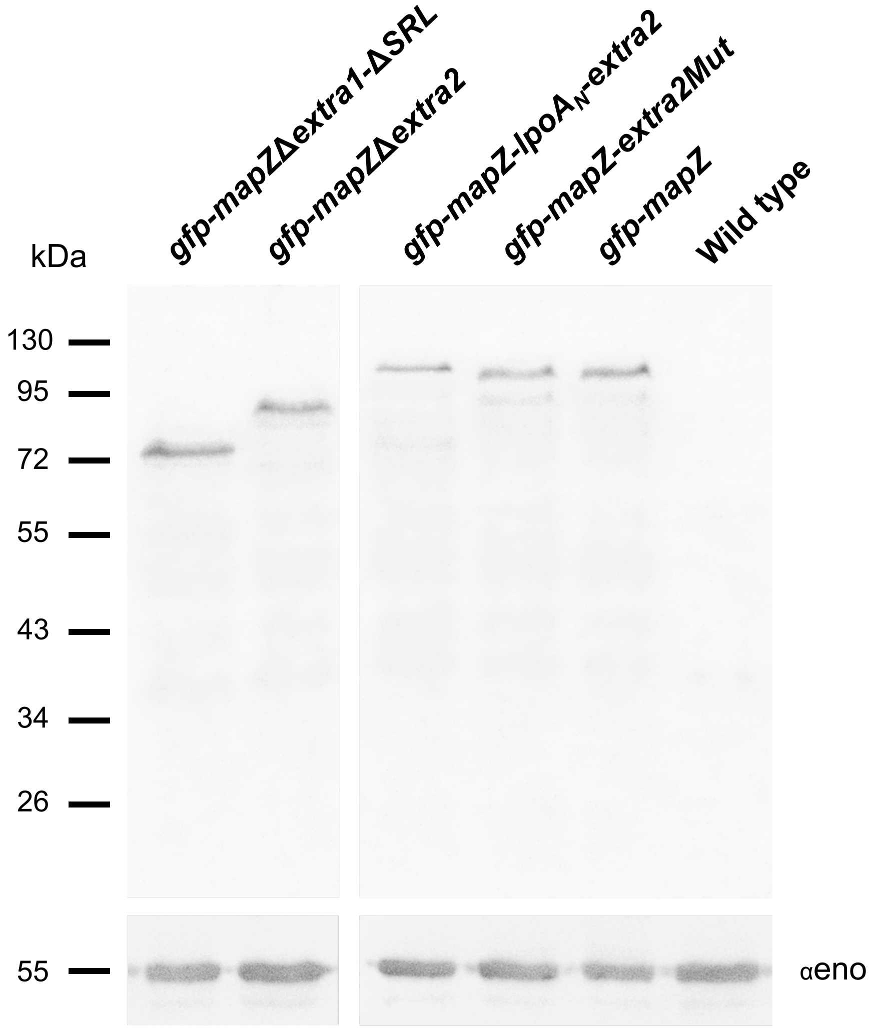

Supplementary Figure 9. Expression of GFP fusions in WT and *mapZ* mutants

Expression of MapZ mutated forms fused to GFP in *gfp-mapZΔextra1-ΔSRL*, *gfp-mapZΔextra2*, *gfp-mapZ-lpoA$_N$-extra2*, *gfp-mapZextra2Mut, gfp-mapZ* and wild-type strains. Cells were grown in THY medium at 37°C to OD_{550} = 0.3. Crude extracts (25 µg) were analyzed by SDS-PAGE, electro-blotted onto a PVDF membrane and probed with anti-GFP antibodies. To estimate the relative quantity of proteins in crude extracts and to compare the different lanes, a second immunoblot analysis, using the enolase as an internal standard was performed. Detection of the enolase in each sample using specific antibodies (αEno, doi: 10.1111/j.1365-2958.2011.07962.x) is presented in the lower part of the Figure.

Supplementary Figure 10

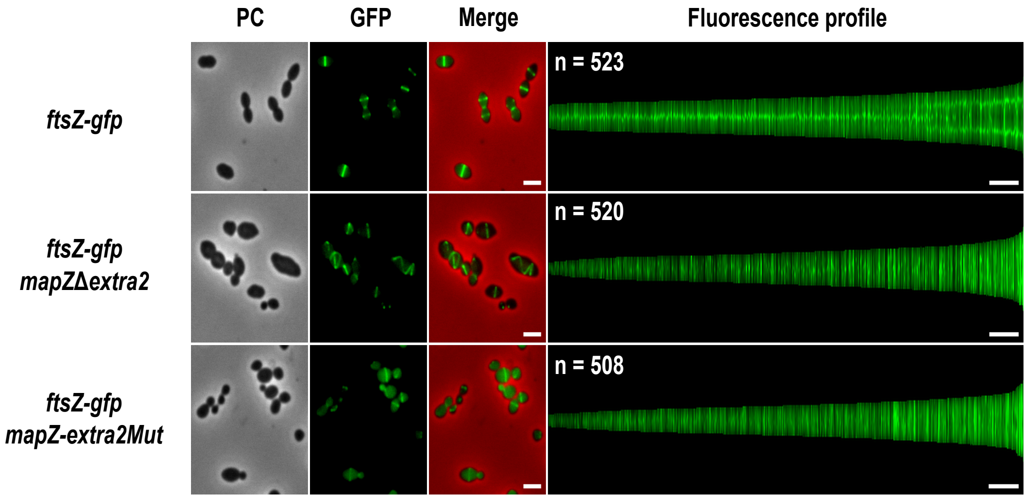

Supplementary Figure 10. Localization of FtsZ-GFP in WT, and *mapZΔextra2* and *mapZ-extra2Mut* cells

Phase contrast (left), GFP fluorescent signal (middle), and overlays (right) between phase contrast (red) and GFP (green) images, as well as the map of FtsZ-GFP fluorescence profiles, are shown. The total integrated fluorescence intensity of each cell (y-axis) is plotted as a function of its cell length (x-axis). Cells are sorted according to increasing cell length from left to right on the later axis. For each fluorescence profile, n indicates the total number of cells analyzed. Scale bars on the microscopy images and fluorescence profiles correspond to 2 μm.

Supplementary Figure 11

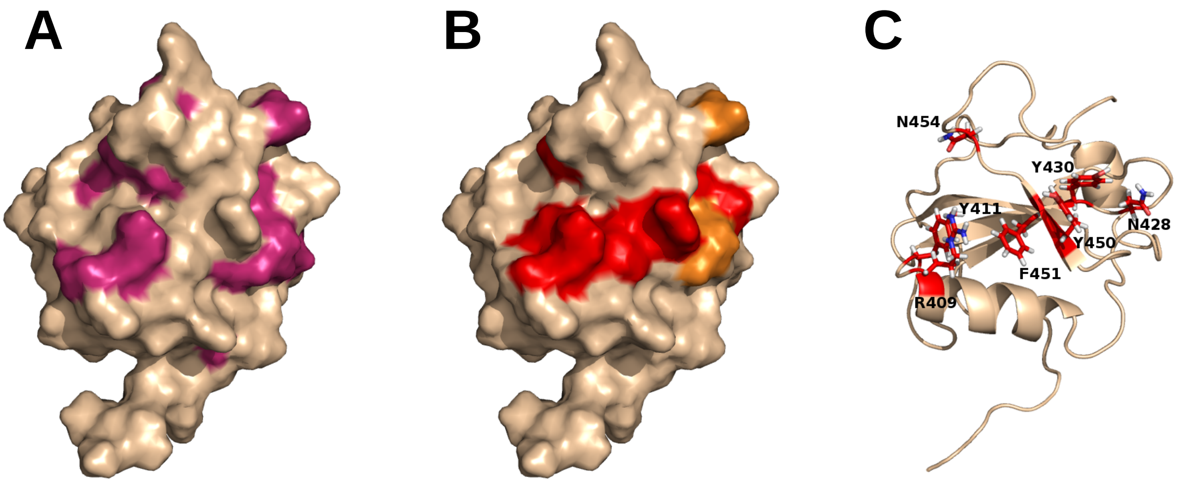

Supplementary Figure 11. The patch of seven conserved surface-exposed amino acids of $MapZ_{extra2}$

Surface of $MapZ_{extra2}$ colored (A) in purple for the highly conserved residues, (B) in red for the seven-residues mutation performed in this study. Deleterious mutations for MapZ folding are indicated in orange in panel B. (C) The seven amino acids of the conserved patch are shown as sticks together with the secondary structure elements of $MapZ_{extra2}$.

Supplementary Figure 12

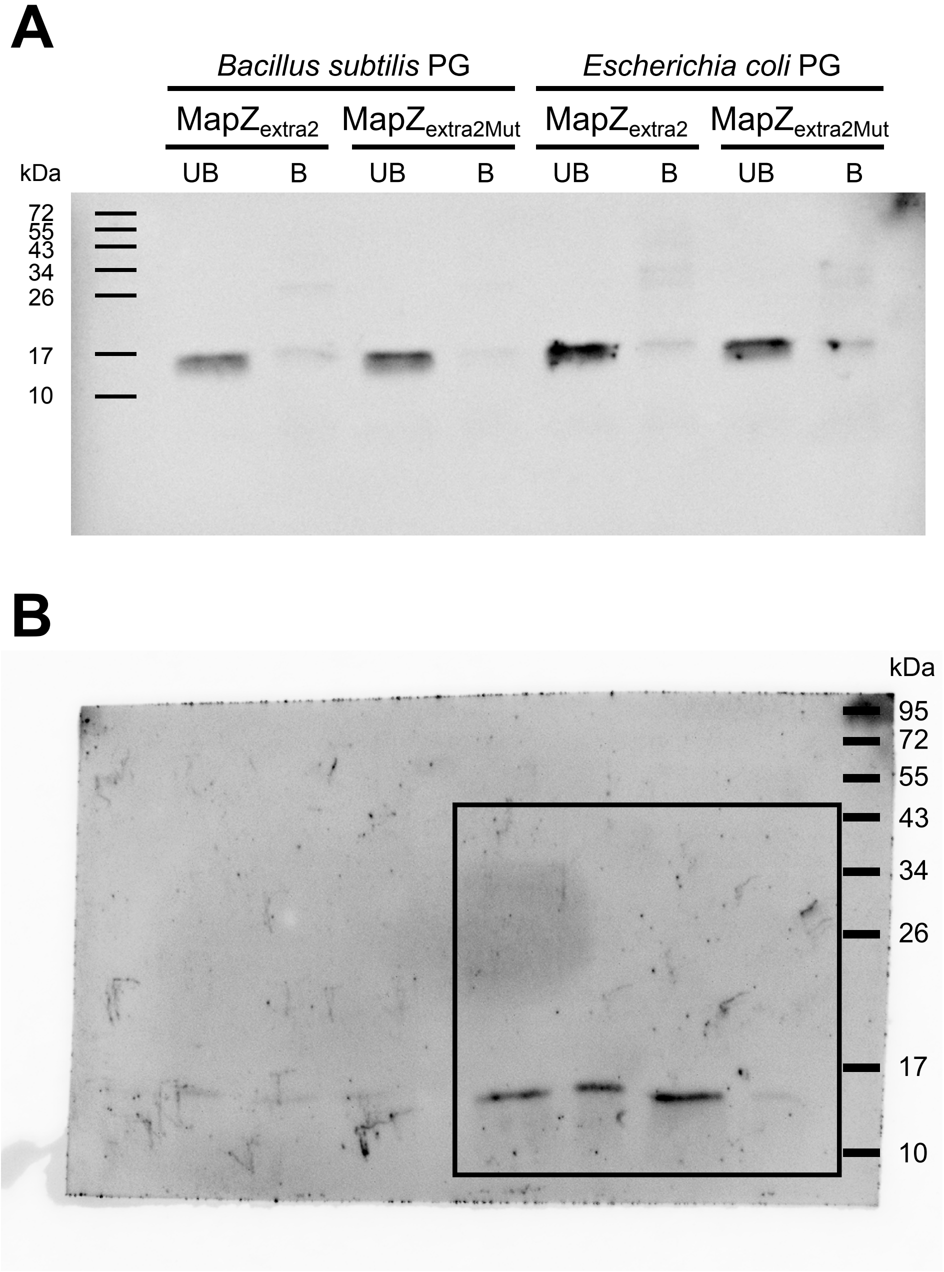

Supplementary Figure 12. Interaction of MapZ$_{extra2}$ with peptidoglycan

(A) Interaction of MapZ$_{extra2}$ and MapZ$_{extra2Mut}$ with peptidoglycan from *Escherichia coli* and *Bacillus subtilis*. The fraction of MapZ$_{extra2}$ or MapZ$_{extra2Mut}$ unbound to peptidoglycan (UB) and bound to cell wall (B) were detected using a mouse anti-histidine-tag antibody. The experiment was made in triplicate. (B) Full blot image corresponding to Figure 6A. The latter is represented by the black rectangle.

SUPPLEMENTARY TABLES

Supplementary Table 1. Strain viability and generation time

Strains	Viability[a] (%)	Generation time[b] (min)
Wild type	100 ± 7	32 ± 0.5
mapZΔextra2	75.6 ± 3.9	42 ± 2.5
mapZ-extra2Mut	82.8 ± 5.5	43 ± 2.5
mapZΔextra1-ΔSRL	66.4 ± 4.8	42.5 ± 1.5
mapZ-lpoA$_N$-extra2	100.9 ± 5.1	31.5 ± 1.5
ΔmapZ	70.3 ± 0.7	48 ± 2

[a] Colony-forming units per milliliter (c.f.u. mL^{-1}) estimated by plating and normalized to that of wildtype strain. Data are shown with s.d. for three independent experiments.
[b] Time required for doubling of the optical density (OD_{550nm}) in liquid culture. Data are shown with s.d. for three independent experiments.

Supplementary Table 2. Strains and plasmids

Strains	Genotype and description	References
***S. pneumoniae* strains**		
R800	*S. pneumoniae* R6 derivative	Gift from J.-P. Claverys, Toulouse, France
WT	R800 *rpsL1*; Str^R	Gift from J.-P. Claverys, Toulouse, France
mapZΔextra2	R800 *rpsL1*, *mapZ*::*mapZ*Δ(*S355-Y464*); Str^R	This study
mapZ-extra2Mut	R800 *rpsL1*, *mapZ*::*mapZ-R409A-Y411A-N428A-Y430F-Y450A-F451L-N454A*; Str^R	This study
mapZΔextra1-ΔSRL	R800 *rpsL1*, *mapZ*::*mapZ*Δ(*Q182-G313*); Str^R	This study
mapZ-lpoA$_N$*-extra2*	R800 *rpsL1*, *mapZ*::*mapZ*[(*Q182-G313*)::*lpoA*(*G28-T256*)]; Str^R	This study
gfp-mapZ	R800 *rpsL1*, *mapZ*::*gfp-mapZ*; Str^R	[4]
gfp-mapZΔextra2	R800 *rpsL1*, *mapZ::gfp-mapZ*Δ(*S355-Y464*); Str^R	This study
gfp-mapZ-extra2Mut	R800 *rpsL1*, *mapZ*::*gfp-mapZ-R409A-Y411A-N428A-Y430F-Y450A-F451L-N454A*; Str^R	This study
gfp-mapZΔextra1-ΔSRL	R800 *rpsL1*, *mapZ*::*gfp-mapZ*Δ(*Q182-G313*); Str^R	This study
gfp-mapZ-lpoA$_N$*-extra2*	R800 *rpsL1*, *mapZ*::*gfp-mapZ*[(*Q182-G313*)::*lpoA*(*G28-T256*)]; Str^R	This study
ftsZ-gfp	R800 *rpsL1*, *ftsZ*::*ftsZ-gfp*; Str^R	[19]
ftsZ-gfp_mapZΔextra2	R800 *rpsL1*, *ftsZ*::*ftsZ-gfp*, *mapZ*::*mapZ*Δ(*S355-Y464*); Str^R	This study
ftsZ-gfp_mapZ-extra2Mut	R800 *rpsL1*, *ftsZ*::*ftsZ-gfp*, *mapZ*::*mapZ-R409A-Y411A-N428A-Y430F-Y450A-F451L-N454A*; Str^R	This study
***E. coli* strains**		
XL1-Blue	*sup*E44 *hsd*R17 *rec*A1 *end*A1 *gyr*A46 *thi rel*A1 lac- F′[*pro*AB + *lac*I^q *lac*ZDM15 Tn10 (Tc^R)]	[5]
BL21(DE3)	F- *omp*T *gal dcm lon hsd*S$_B$(r_B- m_B-) l(DE3 [*lac*I *lac*UV5-T7 gene 1 *ind*1 *sam*7 *nin*5])	[6]
**Plasmids**		
pT7-7-*mapZ*$_{extra}$	pT7-7 derivative, encoding the whole extracellular domain of MapZ, from Gln182 to Tyr464, Amp^R	[4]
pETPhos	pET28 derivative, encoding a His-tag for N-terminal His-tag fusions, Amp^R	[7]
pETPhos-*mapZ*$_{extra1}$	pETPhos derivative, encoding MapZ$_{extra1}$ from Gln182 to Gly313, Amp^R	This study
pETPhos-*mapZ*$_{extra2}$	pETPhos derivative, encoding MapZ$_{extra2}$ from Ser355 to Tyr464, Amp^R	This study
pETPhos-*mapZ*$_{extra2Mut}$	pETPhos derivative, encoding MapZ$_{extra2}$ from Ser355 to Tyr464, with mutations R409A-Y411A-N428A-Y430F-Y450A-F451L-N454A, Amp^R	This study

Supplementary Table 3. List of primers

Purpose	Gene or plasmid	N°[a]	Sequence 5'-3'[b], gene, position[c]
Construction of *S. pneumoniae* strains	*mapZ*	1 up (+)	GTCTAGCCTCTTTAAACGTGG, upstream of *mapZ*, -688
		2 down (-)	GCATCAAGTCATAGCTTTCTGC, downstream of *mapZ*, +2086
		3 GFP (-)	TCCGGATCCCTCGAGTTTATACAATTCATCCATACCATG, *gfp*, +717
		4 GFP (+)	CTCGAGGGATCCGGAATGAGTAAAAAAGACGAAATCG, *mapZ*, +1
		5 Δextra1-ΔSRL/LpoA$_N$ (-)	ACGATAGACATAATAAGCACTG, *mapZ*, +543
		6 Δextra1-ΔSRL (+)	CAGTGCTTATTATGTCTATCGTAGTCGCAGTGAAGTCAATATGGGTC, *mapZ*, +1063
		7 Δextra2 (-)	ACTTCTAGTCTCATTTGAACTAC, *mapZ*, +1062
		8 Δextra2 (+)	GTAGTTCAAATGAGACTAGAAGTTAAGCAGTCGTTACAAAATTCTTTC, *mapZ*, +1393
		9 LpoA$_N$ (+)	CAGTGCTTATTATGTCTATCGTGGCACCCATACTCCCGATCAG, *lpoA*, +82
		10 LpoA$_N$ (-)	GAAGTACTTGTTTGCTGGCTCTTGGTCGAGGCTGGTTTAAACGC, *lpoA*, +768
		11 LpoA$_N$' (+)	AAGAGCCAGCAAACAAGTACTTC, *mapZ*, +940
		12 R409A-Y411A (-)	GGTCTCCAGTGATA**GC**GCCA**GC**TGAACGTGAAGTC, *mapZ*, +1246
		13 N428A-Y430F (-)	GCTTATAGAGGTTGTAA**A**AACCA**GC**GCCGTTAACGATATTG, *mapZ*, +1306
		14 Y450A-F451L-N454A (-)	GACCAGCGCCA**GC**TCCGAC**T**A**G**G**GC**GCCTGTCTTAC, *mapZ*, +1372
Construction of plasmids for protein overexpression in *E. coli*	pETPhos-*mapZ*$_{extra1}$	15 extra1-5' (+)	GGGAATTC*CATATG*CAAGTGGCTCGTTCGACCAAGG, *mapZ*, +544 (*Nde*I)
		16 extra1-3' (-)	TAT*GGATCC*TTAACCAAGACTGATAGCCTTATCTAGC, *mapZ*, +939 (*Bam*HI)
	pETPhos-*mapZ*$_{extra2}$ pETPhos-*mapZ*$_{extra2Mut}$	17 extra2-5' (+)	GGGAATTC*CATATG*AGTCGCAGTGAAGTCAATATGG, *mapZ*, +1063 (*Nde*I)
		18 extra2-3' (-)	TAT*GGATCC*TTAGTAGTCCAAGTCATCCGC, *mapZ*, +1395 (*Bam*HI)

[a] Forward and reverse primers are represented by plus (+) or minus (-), respectively.
[b] For primer pairs 3/4, 5/6, 7/8, 9/10, 5/9, and 10/11, sequences underlined are complementary to each other. The sequences underlined in primers 3 and 4 code for a linker inserted between MapZ and the GFP. For primers 12 to 14, mutated bases are in bold. For primers 15 to 18, restriction sites are italicized and the corresponding restriction enzymes are indicated in brackets.
[c] - and + indicate respectively upstream and downstream positions relative to the ATG codon of the corresponding gene.

SUPPLEMENTARY REFERENCES

1. Landau, M. *et al.* ConSurf 2005: the projection of evolutionary conservation scores of residues on protein structures. *Nucleic Acids Res.* **33**, W299-302 (2005).

2. Dosztanyi, Z., Csizmok, V., Tompa, P. & Simon, I. IUPred: web server for the prediction of intrinsically unstructured regions of proteins based on estimated energy content. *Bioinformatics* **21**, 3433-3434 (2005).

3. Garcia de la Torre, J., Huertas, M. L. & Carrasco, B. HYDRONMR: prediction of NMR relaxation of globular proteins from atomic-level structures and hydrodynamic calculations. *J. Magn. Reson.* **147**, 138-146 (2000).

4. Fleurie, A. *et al.* MapZ marks the division sites and positions FtsZ rings in *Streptococcus pneumoniae*. *Nature* **516**, 259-262 (2014).

5. Bullock, W.O., Fernandez, J. M., Short J. M. A high efficiency plasmid transforming recA *Escherichia coli* strain with beta-galactosidase selection. *Biotechniques* **5**, 376 (1987).

6. Studier, F.W. & Moffatt, B.A. Use of bacteriophage T7 RNA polymerase to direct selective high-level expression of cloned genes. *J. Mol. Biol.* **189**, 113-130 (1986).

7. Canova, M.J., Kremer, L. & Molle, V. pETPhos: a customized expression vector designed for further characterization of Ser/Thr/Tyr protein kinases and their substrates. *Plasmid* **60**, 149-153 (2008).
